# Potential causal factors of CFS/ME: a concise and systematic scoping review of factors researched

**DOI:** 10.1186/s12967-020-02665-6

**Published:** 2020-12-14

**Authors:** Ashley Elizabeth Muller, Kari Tveito, Inger Johanne Bakken, Signe A. Flottorp, Siri Mjaaland, Lillebeth Larun

**Affiliations:** 1 Norwegian Institute of Public Health, Skøyen, PO Box 222, 0213 Oslo, Norway; 2 Journal of the Norwegian Medical Association, Sentrum, PO Box 1152, 0107 Oslo, Norway; 3 Norwegian Directorate of Health, Skøyen, PO Box 222, 0213 Oslo, Norway; 4 University of Oslo, Oslo, Norway

**Keywords:** Etiology, Scoping review, Patient involvement, Chronic fatigue syndrome, Myalgic encephalomyelitis

## Abstract

**Background:**

Chronic fatigue syndrome/myalgic encephalomyelitis (CFS/ME) is understood as a complex condition, likely triggered and sustained by an interplay of biological, psychological, and social factors. Little oversight exists of the field of causal research. This systematic scoping review explores potential causal factors of CFS/ME as researched by primary studies.

**Methods:**

We searched eight databases for primary studies that examined potential causal factors of CFS/ME. Based on title/abstract review, two researchers independently sorted each study’s factors into nine main categories and 71 subordinate categories, using a system developed with input given during a 2018 ME conference, specialists and representatives from a ME patient advocacy group, and using BMJ Best Practice’s description of CFS/ME etiology. We also extracted data related to study design, size, diagnostic criteria and comparison groups.

**Results:**

We included 1161 primary studies published between January 1979 and June 2019. Based on title/abstract analysis, no single causal factor dominated in these studies, and studies reported a mean of 2.73 factors. The four most common factors were: immunological (297 studies), psychological (243), infections (198), and neuroendocrinal (198). The most frequent study designs were case–control studies (894 studies) comparing CFS/ME patients with healthy participants. More than half of the studies (that reported study size in the title/abstract) included 100 or fewer participants.

**Conclusion:**

The field of causal hypotheses of CFS/ME is diverse, and we found that the studies examined all the main categories of possible factors that we had defined a priori. Most studies were not designed to adequately explore causality, rather to establish hypotheses. We need larger studies with stronger study designs to gain better knowledge of causal factors of CFS/ME.

## Background

CFS/ME is a condition characterized by prolonged, significant, and sometimes disabling exhaustion and post-exertion malaise, accompanied by symptoms such as generalized pain, sleep disorders, and cognitive problems [13, 24, 28]. Fatigue can be aggravated by physical and mental exertion, and does not decrease after rest [22, 28]. Rather than a single cause, it is likely that multiple biological, psychological and/or social factors may predispose, trigger, and maintain this condition [11]. Identifying potential causal factors is imperative to understanding CFS/ME and to developing more effective treatments and prevention.

The vastness of the research field on etiological factors reflects the variety of causal hypotheses [19], and in the past two years alone, numerous systematic reviews have been published examining distinct causal factors such as immunological [12, 16, 29], metabolic [15, 23], circulatory [27], and epigenetic factors [3]. In 2016, the Research Council of Norway selected the CFS/ME field as the pilot field for a research priority setting partnership strategy, in which patients, next of kin, and related organizations prioritized research questions for commissioned research [14]. When topics for CFS/ME-related research proposals were requested [31], patients and next of kin submitted more than 700 submissions. Sixty percent of their submissions involved questions about causal factors. At the suggestion of the Research Council of Norway, we conducted a systematic scoping review of primary studies that examined predisposing, triggering and maintaining factors to CFS/ME.

## Methods

A systematic scoping review presents an overview of research relating to a specific topic or question, without producing a summary answer to that question or assessing the methodological quality of primary studies [4, 33]. This method was well-suited to this review’s exploratory and descriptive aims. Following Arksey and O’Malley’s framework for scoping reviews, we collaborated with a working group from the Norwegian ME Association to develop the research question as well as inclusion and exclusion criteria, and we published a protocol in Norwegian [25]. We included all primary studies, of any design besides case reports, that examined potential predisposing, triggering, and maintaining factors in patients with CFS/ME. We searched eight databases in June 2019 with free text words (in title and summary) and controlled terms for the patient group, combined with free text words and terms for cause/risk. We had no restrictions relating to year of publication or language. Additional file 1: Appendix S1 contains an overview of all databases searched as well as the search strategy for OVID. The PRISMA Checklist for Scoping Reviews is available in Additional file 2: Appendix S2.

Two researchers independently screened titles/abstracts for inclusion using Covidence software [34]. In the event of disagreement or uncertainty, we consulted a third researcher. Using EPPI Reviewer [32], one researcher extracted data from titles/abstracts on diagnostic criteria, comparison group, study design, number of participants, and the region where the study was conducted (Table 1). We did not retrieve the full texts of the included studies, and we based the data extraction on the titles and abstracts only.

**Table 1:** Data categories extracted from titles/abstracts

Abstract	Diagnostic criteria	Number of participants
Yes	Not reported	≤ 30
No	CDC 1988 [21]	31–100
WHO Region	CDC/Fukada 1994 [18]	101–1000
Eastern Mediterranean region	Canadian 2003 [9]	≥ 1,001
Not reported	International consensus criteria [10]	Etiological factors (see Additional file 3: Appendix S3 for the complete categorization scheme)
African region	Arabic Scale of Chronic Fatigue Syndrome [1]	Infections
European region	Other	Immunological
Region of the Americas	Study design	Neuroendocrinal/hormonal/metabolic
South-East Asian region	Not reported	Genetic/epigenetic
Western Pacific region	Case–control	Circulatory
Multiple	Cross-sectional	Gastrointestinal
Comparison group	Cohort (prospective, retrospective)	Neurobiological
Not reported	Other	Psychological/psychosocial/socioeconomic
Type unspecified		Other factors
Non-patient group		
Other patient group		
None		

The seven categories of data extracted from titles/abstracts of included studies. The full categorization scheme, and results, of Etiological factors are presented in Additional file 3: Appendix S3

**Fig. 1:**
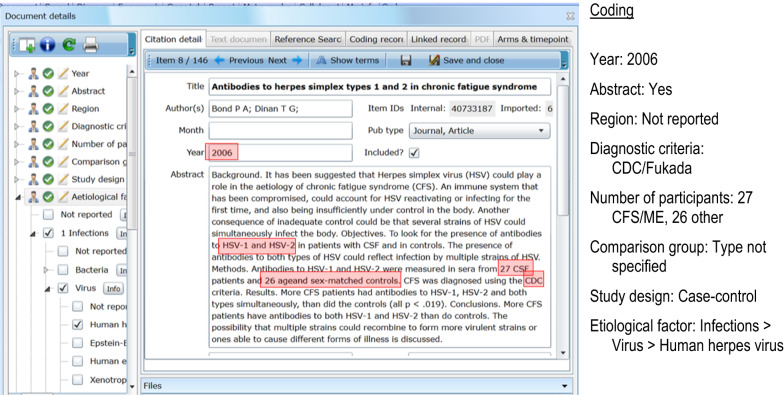
An example of a fully coded study that reports the diagnostic criteria used, number of participants, study design, and one etiological factor

Two researchers independently categorized every potential causal factor using a system of nine major categories, 48 sub-categories, and 23 further subordinate categories, as specified in a codebook. We developed this coding system based on the description of CFS/ME in BMJ Best Practice [6], and supplemented with input from an ME conference in 2018, as well as from specialists and representatives from the ME Association. Figure 1 is a screenshot of an abstract in EPPI Reviewer, annotated to display coding.

The nine causal categories were *infections, immunological*, *neuroendocrinal/hormonal/metabolic*, *genetic/epigenetic*, *circulatory*, *gastrointestinal, neurobiological*, *psychological*/*psychosocial/socioeconomic* and *other factors.* If a study reported multiple factors, we coded all factors, but each factor was mutually exclusive (that is, two categories were never required to describe one factor). Each main category had up to nine sub-categories. For example, we divided *infections* into *bacteria*, *viruses*, *fungi*, *parasites*, *other* and *not reported*. Most sub-categories had further subordinate categories; for example, *viruses* were further categorized *human herpesvirus, Epstein–Barr virus, xenotropic murine leukemia virus, hepatitis virus*, *other*, and *not reported* (Additional file 3: Appendix S3). We developed and refined the codebook continually during the first half of the project, with particular attention to patterns within *other* subordinate categories, such that common factors coded therein would be extracted and receive their own codes.

## Results

**Fig. 2:**
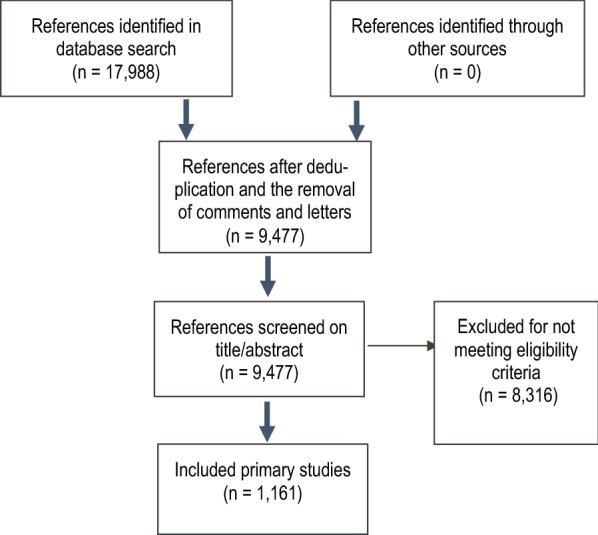
Study flow diagram. A flow chart of the 9477 unique studies retrieved from database searches, of which 1161 met inclusion criteria after title/abstract review

Figure 2 displays the results of the search and screening process. In total, 1161 studies were included (Additional file 4: Appendix S4).

### Characteristics of studies

**Fig. 3:**
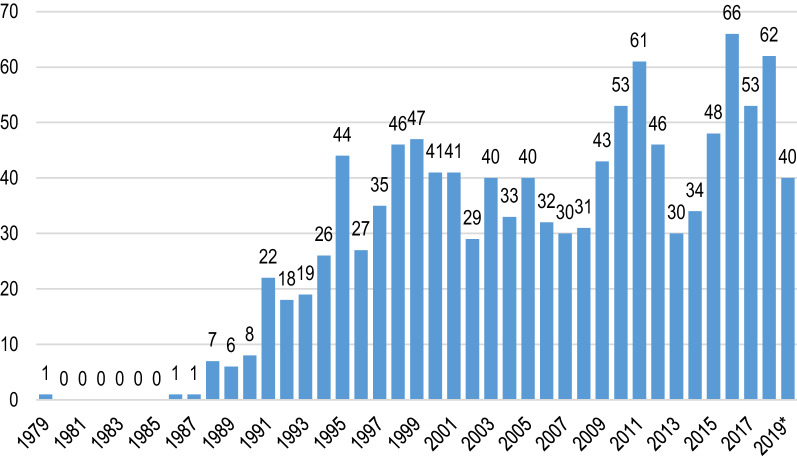
Studies published per year, 1979–June 2019. *Published before June 2019. The number of studies published per year has increased steadily since the late 1980’s, with at least 48 studies published yearly 2015–2019, and 40 published in the first half of 2019

The number of published studies per year increased from one in 1979 to 40 from January to June 2019 (Fig. 3).

We extracted all data from the studies’ titles and abstracts. Of 1161 studies, 68 were missing abstracts. Only 217 of the studies reported which diagnostic criteria were used in the titles/abstracts; 169 of these used the 1994 CDC/Fukada criteria [18]. Regarding study design, most studies (889) were case–control studies, in which CFS/ME patients were compared to some type of control or comparison group, at one point in time. There were 115 cross-sectional studies without a comparison or control group. A total of 67 studies were prospective or retrospective cohort studies. Fourteen studies fell into the category of "other" study design, which included twin studies, registry studies, and population-based screening studies. In 69 cases, study design was not reported in titles/abstracts.

A total of 679 prospective, retrospective, case–control, or "other" designs included comparison groups, and most used healthy/non-patient comparisons (61 to 66% of studies within each study design). Only 226 studies used other patient groups as comparisons. 181 studies used a comparison group but did not specify which type, and 73 did not report on comparison groups in titles/abstracts.

Study size varied considerably: in 117 studies, there were fewer than 30 participants, 440 had between 30–100 participants, 289 contained 101–1000 participants, and 31 contained more than 1000 participants. Cohort and "other" study designs were largest. Finally, only 170 studies stated in titles/abstracts the country in which they were conducted. Nearly half (87) were European, while 56 were conducted in North or South America.

### Potential causal factors examined

Most studies examined several different potential causal factors (mean 2.73; range from 1 to 5, out of a possible 9). The most frequently studied categories were *immunological* (272 studies), *psychological*/*psychosocial/socioeconomic* (243 studies), *infections* (198 studies) and *neuroendocrinal/hormonal/metabolic* (197 studies).

**Fig. 4:**
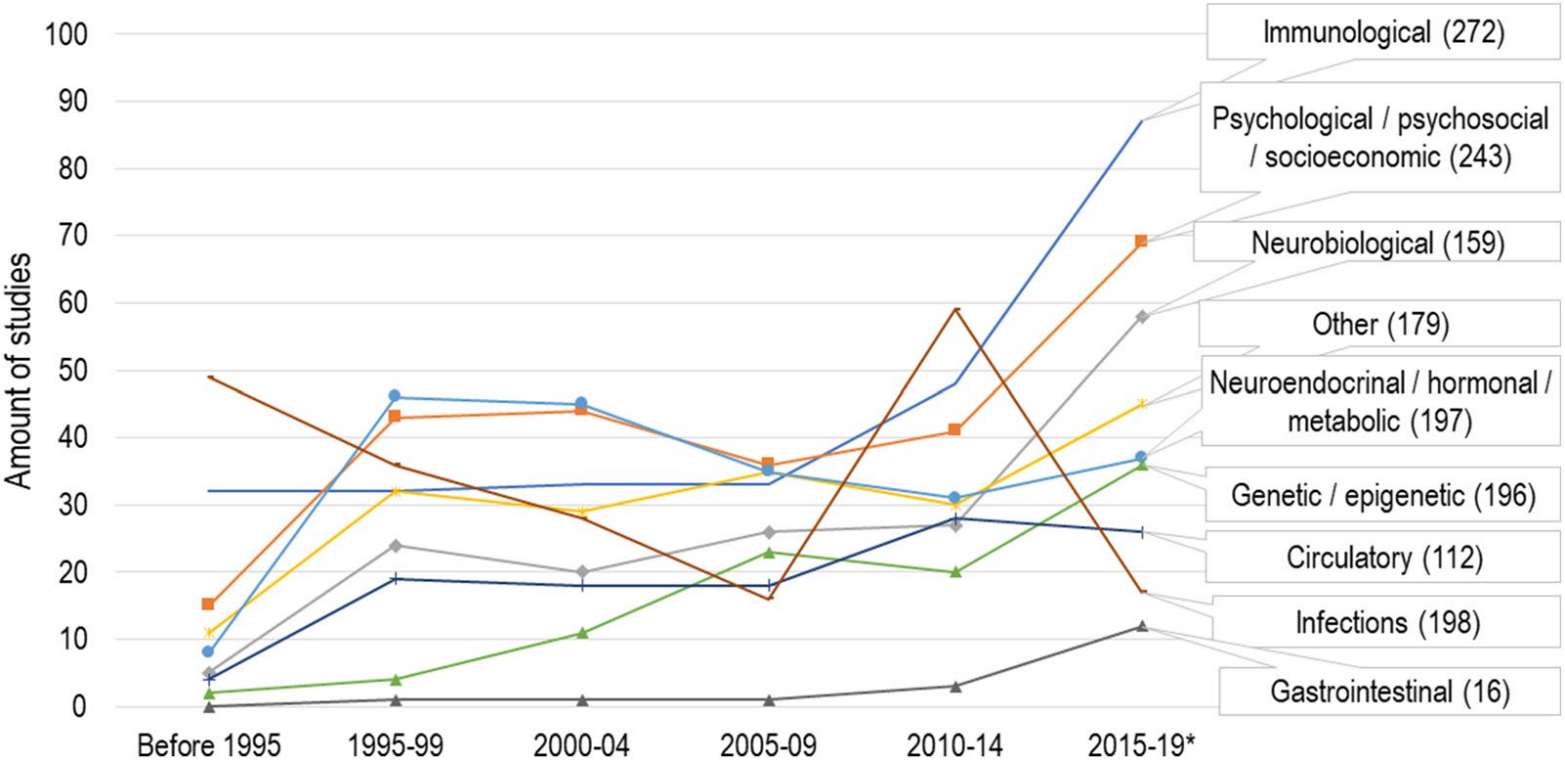
The number of studies researching each potential causal factor in 5-year periods. *Number published before June 2019. Most potential causal factors, with the exception of infections, have been explored by an increasing number of studies since 2010. Some studies involved multiple factors and were counted in each category

No single causal factor has dominated research in the field (Fig. 4). The number of studies on infection factors varied the most: it was the category with the largest number of studies before 1995 as well as in 2010–2014, but in the recent period of 2015–June 2019, there was a marked decrease. For all other categories, there was an increase in the number of studies from 2010 to 2019.

Additional file 3: Appendix S3 shows the number of studies in each category. For example, in the category of infections, there were 158 studies on CFS/ME related to viruses, 35 about bacteria, three about parasites, one about fungal infections, six about other infections, and six that did not report the type of virus. In the sub-category of viruses, there were 53 studies on the Epstein–Barr virus, 47 about human herpes virus, 29 about xenotropic murine leukemia-related virus, five about other humane endogenous retroviruses, four about hepatitis virus, 70 about other viruses, and four that did not report type of virus.

## Discussion

This systematic scoping review included 1161 primary studies on possible causal factors of CFS/ME, and we extracted data from titles and abstracts. Immunological, psychological, infections, and neuroendocrine factors have been most frequently studied. In most categories, there was a steady increase in the number of studies from 1994 and a slightly stronger increase from 2010 to 2019. Many studies were not limited to a single factor or causal hypothesis, but explored numerous factors, from environmental factors to genes. We did not find one dominant causal factor studied, and included studies reported a mean of nearly three different factors, which we interpret as a healthy diversity in the research field. As the number of published articles has increased steadily from 1979–2019, the variation of explored factors has also remained high. This indicates a great deal of uncertainty in the field, but the broad interest in diverse possible causal factors is positive.

A 2008 review included 11 studies that examined multiple possible “risk factors or prognostic flags” [20]. Our scoping review included 1161 studies, and although our scope was larger than the 2008 review, there is a clear increase in research in the field. However, our findings echo the conclusions of numerous recent reviews [3, 12, 15, 16, 23, 27, 29], namely, that our confidence in the evidence is too low to draw conclusions about causal factors. This is due to the preponderance of relatively weak study designs. For most questions regarding etiology, we must rely on observational studies, as experimental trials randomly exposing people to hypothesized causal factors are rarely possible to conduct, and even if technically possible, would be unethical. Cross-sectional studies with or without comparisons or controls can provide evidence on correlations and help develop theories or hypotheses of causality, but are ill-suited to verify them. Most of the studies included were designed so that they were not suitable to detect causality, although many claimed this as their purpose. Even though studies might identify a correlation between a factor and a diagnosis, we cannot know if it is a signal of a causal factor, or a consequence of the diagnosis. We found no increase in the number of prospective, longitudinal studies being published over time. Prospective cohort studies following individuals that are exposed versus not exposed will provide stronger evidence on etiology than case control studies and retrospective cohort studies, but unmeasured confounding will inevitably lead to risk of bias, as in all observational studies.

In addition, more than half of the studies (that reported study size) included fewer than 100 participants, and around 60% of all studies compared CFS/ME patients with healthy controls—potentially inappropriate comparisons. If effect sizes are small, such small studies likely lack the statistical power to reveal differences between CFS/ME patients and other groups. However, determining statistical power of primary studies will only be possible with a full systematic review that examines studies in full-text. While it may be difficult to recruit enough patients with CFS/ME for studies, researchers need to prioritize prospective cohort studies with a sufficient number of participants. When enough data is collected to allow for advanced statistical techniques, sufficient comparison of individuals with different risks, exposure, and genetic variables is possible [26, 30]; propensity score matching, for example, has been recently utilized in studies of other rare diseases and exposures [2].

An additional research challenge is the unambiguous diagnosis of CFS/ME using standard criteria. A systematic review from 2014 identified 20 different sets of diagnostic criteria [7]. CDC 1994/Fukuda criteria [18] were the most commonly used in that review, as in ours. This review did not find any evidence that some of the diagnostic criteria sets could identify conditions more likely with e.g. a specific organic etiology. In our sample based on titles/abstracts, only 18% of studies reported which diagnostic criteria had been used. Some countries have registers of CFS/ME patients that are voluntarily organized and use combinations of self-report and symptom-based criteria (e.g. [8], while Norway and others use diagnostic codes (ICD-10, G.93.3) reported in broader nationwide registries [5]. As it is unclear if the type of diagnostic criteria or case definitions used influences the findings of primary studies, the way the patients are diagnosed should be clearly stated and, ideally, standardized in future studies [17].

## Strengths and limitations

We searched in eight databases and used a broad and sensitive search strategy. One strength of the study is that a CFS/ME patient advocacy group helped formulate the research questions and provided substantial input to the data extraction form and the causal factor categorization system. An overall strength of scoping reviews is that they provide a descriptive overview of existing research and knowledge gaps. A clear limitation of this review is that we extracted data from titles/abstracts only, and not from full-texts. While this was a pragmatic decision based on resources available, reading full texts would likely have provided us more information about study size, region, and comparison groups. It is important for readers to note that much of the data we report as “missing” from the title/abstract level may have been reported in full text. As we did not aim to synthesize the results of the studies, we think that the data extracted from the titles/abstracts provides the intended descriptive overview of the categories of causal factors that have been studied, and the study designs that have been used.

## Conclusion

We saw a large variety of causal factors explored, although a precipitous decline in research on infections as causal factors in the past five years. It is problematic that research into causal factors of CFS/ME used different study designs, diagnostic criteria, control groups, and methods in general. Our scoping review shows that larger studies with stronger designs are needed. We hope to see more well-designed prospective cohort studies in the future, particularly as post-covid-19 fatigue—and the potential risk for developing CFS/ME after infection with Sars-CoV-2—is explored.

## Supplementary information

**Additional file 1: Appendix S1.** Search strategies and the databases we searched in June 2019 and December 2019.

**Additional file 2: Appendix S2.** Completed Preferred Reporting Items for Systematic reviews and Meta-Analyses extension for Scoping Reviews (PRISMA-ScR) Checklist.

**Additional file 3: Appendix S3.** The potential causal factors researched by 1,161 included primary studies, divided into nine main categories and 48 sub-categries. An additional 23 further subordinate categories are not shown, and are available by the authors upon request.

**Additional file 4: Appendix S4.** A list of all included 1,161 primary studies sorted alphabetically by main potential causal factor(s) researched.

## Data Availability

Secondary data may be made available by the corresponding author upon request.

## Abbreviation

CFS/ME: Chronic fatigue syndrome/myalgic encephalomyelitis
