# Supplementary material for: Potential causal factors of CFS/ME: a concise and systematic scoping review of factors researched
Source: J Transl Med. 2020 Dec 14;18:484. doi: 10.1186/s12967-020-02665-6 (PMC7734915; doi:10.1186/s12967-020-02665-6)
Supplement: Supplementary file 1 — Additional file 1: Appendix S1. Search strategies and the databases we searched in June 2019 and December 2019. [file 12967_2020_2665_MOESM1_ESM.docx]

### Appendix 1 Database and search strategy

We searched in the following databases in June 2019:

• AMED (Allied and Complementary Medicine) (Ovid) (years: 1985 – present)

• APA PsycINFO (Ovid) (years: 1806 – present)

• CINAHL (EBSCO) (years: 1981 – present)

• Cochrane Central Register of Controlled Trials (Wiley)

• Embase (Ovid) (years: 1975 – present)

• Ovid MEDLINE(R) and Epub Ahead of Print, In-Process & Other Non-Indexed Citations, Daily and Versions(R) (years: 1946 – present)

• Web of Science (Clarivate Analytics) (years: 1900 – present)

In December 2019, we searched:

• Epistemonikos (Epistemonikos Foundation)

Search strategy Ovid (AMED, Embase, MEDLINE og PsycINFO)

| 1 | Fatigue Syndrome, Chronic/ use ppezv or *chronic fatigue syndrome/ use oemezd or Chronic Fatigue Syndrome/ use psyh or Fatigue syndrome chronic/ use amed |
| --- | --- |
| 2 | (chronic fatigue syndrome or myalgic encephal* or "CFS/ME" or "ME/CFS" or systemic exertion intolerance).tw,kw,id,hw. |
| 3 | 1 or 2 |
| 4 | (etiolog* or aetiol* or correlat* or risk* or cause* or causation* or causal* or etiopatho* or aetiopatho* or physiopath* or pathogen* or ((enabl* or predispos* or reinforc* or precipit* or protect* or immun* or neuroendocrin* or neuro-endocrin* or metabolic or genetic or epigenetic or circulatory or gastrointestinal or gastro-intestinal or neurobiologic* or psychological or psychosocial or psycho-social or socioeconomic or socio-economic) adj3 factor*) or epidemiol* or determinant* or predict* or correspond* or associat* or originat* or correlat* or trigger* or induc* or predispose* or mechanism* or relation* or comorbidit*).tw,kw,kf,id,hw. |
| 5 | factor*.ti. |
| 6 | (exp Causality/ or exp Cohort Studies/ or exp Epidemiologic Studies/) use ppezv or ((case control study/ or cohort analysis/ or longitudinal study/ or follow up/ or prospective study/ or retrospective study/ or observational study/ or causality/ or etiology/ or correlation analysis/ or risk factor/ or protection/) use oemezd) or ((etiology/ or causality/ or risk factors/ or protective factors/ or Cohort analysis/ or longitudinal studies/ or exp prospective studies/ or exp followup studies/ or exp retrospective studies/ or ("0430" or "0450" or "0451" or "0453").md.) use psyh) or ((etiology/ or risk factors/ or case control studies/ or cohort studies/ or prospective studies/ or retrospective studies/) use amed) |
| 7 | (cohort* or case control*).mp. or ((longitud* or prospective* or retrospective* or follow-up or followup or epidemio* or observation*) adj3 (study or studies or analys* or evaluation*)).tw. |
| 8 | or/4-7 |
| 9 | 3 and 8 |
| 10 | ((virus* or viral or bacteria* or infection* or parasite* or personalit* or lifestyle* or mononucleos* or stress or influenza or common cold or trauma* or inflammation* or psychiatr* or vaccin* or immun* or post-infect* or postinfect* or post-viral or postviral or cytokine* or neuroendocrin* or neuro-endocrin* or metabolic or genetic* or epigenetic* or circulatory or gastrointestinal or gastro-intestinal or neurobiologic* or biologic* or psychological or psychosocial or psycho-social or socioeconomic or socio-economic) and (chronic fatigue syndrome or myalgic encephal* or "CFS/ME" or "ME/CFS" or systemic exertion intolerance)).ti. |
| 11 | Fatigue Syndrome, Chronic/ah, bl,cf,ci, cn,en,ep,et, ge, im, me,mi, ps, pa, ph, pp, px, vi use ppezv or chronic fatigue syndrome/cn,ep,et use oemezd |
| 12 | 9 or 10 or 11 |
| 13 | animal/ not human/ use ppezv [MEDLINE] |
| 14 | ((exp animal/ or nonhuman/) not exp human/) use oemezd [Embase] |
| 15 | (conference paper or conference review or conference abstract).pt. use oemezd [Embase] |
| 16 | 12 not (13 or 14 or 15) |

| Codes and symbols in OVID databases | |
| --- | --- |
| "xxx"/ | Subject heading from database thesaurus (MeSH, Emtree, American Psychological Association's Thesaurus of Psychological Index Terms) |
| exp "xxx"/ | Expand results to include records about the narrower, more specific terms. |
| ppezv | Code for the MEDLINE database segment searched. (Ovid MEDLINE(R) and Epub Ahead of Print, In-Process & Other Non-Indexed Citations, Daily and Versions(R) 1946 to June 19, 2019) |
| oemezd | Code for the Embase database segment searched. (Embase 1974 to 2019 June 19) |
| psyh | Code for the APA PsycINFO database segment searched. (PsycINFO 1806 to June Week 3 2019) |
| amed | Code for the AMED database. (AMED (Allied and Complementary Medicine) 1985 to June 2019) |
| adjn | The defined adjacency operator (ADJn) retrieves records that contain search terms within a specified number (n-1) of words from each other in any order (stop-words included). |
| * | Truncation |
| .tw | The Text Word (TW) index is an alias for all of the fields in a database which contain text words and which are appropriate for a subject search. |
| .kf | MEDLINE: The Keyword Heading Word (KF) index allows you to retrieve every Keyword Heading assigned by authors that include a particular word |
| .kw | Embase: This Keyword field (KW) contains keywords defined by the author of the article. |
| .pt | Publication Type |
| .hw | PsycINFO: The Heading Word (HW) index allows you to retrieve every subject heading that includes a particular word by searching a single word in the Subject Heading (SH) field. |
| .id | PsycINFO: The Key Concepts (ID) field concisely summarizes a document's subject content. Indexers use the Key Concepts to supplement Subject Headings. |
| .md | PsycINFO: The Methodology (MD) field contains the specific kind of methodology used in a research study. |
| Line11 | Fatigue Syndrome, Chronic/ah, bl, cf, ci, cn, en, ep, et, ge, im, me, mi, ps, pa, ph, pp, px, vi use ppezv  ah = anatomy and histology  bl = blood  cf= cerebrospinal fluid  ci = chemically induced  cn = congenital  en = enzymology  ep = epidemiology  et = etiology  ge = genetics  im = immunology  me = metabolism  mi = microbiology  ps = parasitology  pa = pathology  ph = physiology  pp = physiopathology  px = psychology |
| Line 11 | Chronic Fatigue Syndrome/cn, ep, et use oemezd  cn=congenital disorder  ep = epidemiology  et = etiology |
