# Supplementary material for: Potential causal factors of CFS/ME: a concise and systematic scoping review of factors researched
Source: J Transl Med. 2020 Dec 14;18:484. doi: 10.1186/s12967-020-02665-6 (PMC7734915; doi:10.1186/s12967-020-02665-6)
Supplement: Supplementary file 3 — Additional file 3: Appendix S3. The potential causal factors researched by 1,161 included primary studies, divided into nine main categories and 48 sub-categries. An additional 23 further subordinate categories are not shown, and are available by the authors upon request. [file 12967_2020_2665_MOESM3_ESM.docx]

| **Categories and sub-categories of potential causal factors** | **N** | **% of studies** |
| --- | --- | --- |
| Immunological | 272 | 23.4 |
| - Not reported | 4 |  |
| - Humoral immunity | 96 |  |
| - Cellular immunity | 187 |  |
| - Vaccines | 16 |  |
| - Allergy | 11 |  |
| - Other | 23 |  |
|  |  |  |
| Psychological/psychosocial/socioeconomic | 243 | 20.9 |
| - Not reported | 2 |  |
| - Mental illness | 103 |  |
| - Psychosocial problems | 50 |  |
| - Trauma | 24 |  |
| - Socioeconomic | 11 |  |
| - Personality trait | 48 |  |
| - Other | 115 |  |
|  |  |  |
| Infections | 198 | 17.1 |
| - Not reported | 6 |  |
| - Bacteria | 35 |  |
| - Virus | 158 |  |
| - Yeast infection | 1 |  |
| - Parasites | 3 |  |
| - Other | 6 |  |
|  |  |  |
| Neuroendocrinal/hormonal/metabolic | 197 | 16.5 |
| - Hypothalamus-pituitary-adrenal axis | 83 |  |
| - Disturbance to amino acid and nitrogen metabolism | 7 |  |
| - Glucose/gluconeogenesis | 4 |  |
| - Neurotransmitters | 17 |  |
| - Mitochondrial | 21 |  |
| - Other | 101 |  |
|  |  |  |
| Other | 179 | 15.8 |
| - Hypersensitivities | 14 |  |
| - Organic | 3 |  |
| - Stress | 21 |  |
| - Hematology | 13 |  |
| - Other | 96 |  |
|  |  |  |
| Neurobiological | 159 | 13.7 |
| - Not reported | 2 |  |
| - Dysregulation of myelination | 7 |  |
| - Cerebrovascular dysautoregulation | 5 |  |
| - Neuroinflammation | 6 |  |
| - Neuroanatomic region affected | 77 |  |
| - Sleep disturbances | 51 |  |
| - Pain | 8 |  |
| - Autonomic nervous system | 20 |  |
| - Other | 29 |  |
|  |  |  |
| Circulatory | 112 | 9.6 |
| - Not reported | 3 |  |
| - Cereral blood perfusion/fow | 32 |  |
| - Orthostatic instability | 42 |  |
| - Other | 53 |  |
|  |  |  |
| Genetic/epigenetic | 96 | 8.3 |
| - Not reported | 10 |  |
| - Specified | 90 |  |
|  |  |  |
| Gastointestinale | 16 | 1.4 |
| - Not reported | 2 |  |
| - Microbiotic | 4 |  |
| - Other | 11 |  |
|  |  |  |
| Not reported | 6 | 0.5 |
| Note: Nine categories and 48 sub-categories are shown, while an additional 23 further subordinate categories are not shown. These are available by the authors upon request. | | |
